# Whole genome prediction and heritability of childhood asthma phenotypes

**DOI:** 10.1002/iid3.133

**Published:** 2016-11-28

**Authors:** Michael J. McGeachie, George L. Clemmer, Damien C. Croteau‐Chonka, Peter J. Castaldi, Michael H. Cho, Joanne E. Sordillo, Jessica A. Lasky‐Su, Benjamin A. Raby, Kelan G. Tantisira, Scott T. Weiss

**Affiliations:** ^1^ Channing Division of Network MedicineBrigham and Women's Hospital and Harvard Medical SchoolBostonMassachusetts

**Keywords:** Childhood asthma, heritability, longitudinal lung function patterns, polygenic prediction, whole‐genome prediction

## Abstract

**Introduction:**

While whole genome prediction (WGP) methods have recently demonstrated successes in the prediction of complex genetic diseases, they have not yet been applied to asthma and related phenotypes. Longitudinal patterns of lung function differ between asthmatics, but these phenotypes have not been assessed for heritability or predictive ability. Herein, we assess the heritability and genetic predictability of asthma‐related phenotypes.

**Methods:**

We applied several WGP methods to a well‐phenotyped cohort of 832 children with mild‐to‐moderate asthma from CAMP. We assessed narrow‐sense heritability and predictability for airway hyperresponsiveness, serum immunoglobulin E, blood eosinophil count, pre‐ and post‐bronchodilator forced expiratory volume in 1 sec (FEV_1_), bronchodilator response, steroid responsiveness, and longitudinal patterns of lung function (normal growth, reduced growth, early decline, and their combinations). Prediction accuracy was evaluated using a training/testing set split of the cohort.

**Results:**

We found that longitudinal lung function phenotypes demonstrated significant narrow‐sense heritability (reduced growth, 95%; normal growth with early decline, 55%). These same phenotypes also showed significant polygenic prediction (areas under the curve [AUCs] 56% to 62%). Including additional demographic covariates in the models increased prediction 4–8%, with reduced growth increasing from 62% to 66% AUC. We found that prediction with a genomic relatedness matrix was improved by filtering available SNPs based on chromatin evidence, and this result extended across cohorts.

**Conclusions:**

Longitudinal reduced lung function growth displayed extremely high heritability. All phenotypes with significant heritability showed significant polygenic prediction. Using SNP‐prioritization increased prediction across cohorts. WGP methods show promise in predicting asthma‐related heritable traits.

## Introduction

Asthma is a major chronic childhood disease (9% prevalence) in the USA 1, 2. It is a heterogeneous disease, with varying outcomes and clinical courses, ranging from chronic airway obstruction 3 to the remission of symptoms entirely 4. Forecasting such diverse clinical outcomes and disease phenotypes is an important goal of personalized medicine, and one that may be achieved in part with recent advances in whole‐genome prediction (WGP) 5, wherein a patient's entire set of single nucleotide polymorphisms (SNPs) can be used to predict outcomes of interest. The extent to which WGP can be successfully applied to features of asthma and asthma management have yet to be thoroughly explored.

Adult asthma is associated with an accelerated rate of decline in forced expiratory volume in 1 sec (FEV_1_) 6, 7, and childhood asthmatics have been shown to have lower lung function than non‐asthmatics 8. For asthmatics, reduced lung function leads to several adverse outcomes. Reduced lung function has been associated with increased incidence of asthma attacks among asthmatics 4; children with untreated asthma have shown loss of lung growth velocity 9; and low lung function has predicted late‐onset asthma in unaffected adults 10. Reduced early‐life lung function and a childhood asthma diagnosis independent of lung function have been linked to later decline in lung function 11, sometimes leading to chronic airway obstruction (CAO) and also chronic obstructive pulmonary disease (COPD) 12. Furthermore, genetic risk factors for low FEV_1_ and low FEV_1_ to forced vital capacity ratio (FEV_1_/FVC) have been shown to also be associated with greater risk of COPD 13.

The degree to which genetic prediction is possible is bounded by the heritability of the trait to be predicted 14. Asthma and associated traits are heritable, with twin studies establishing the genetic heritability of asthma incidence to be between 50% and 60% 15, and a recent twin‐study meta analysis determined asthma heritability to be 53% 16. Lung function is also heritable 17, 18, with FEV_1_ heritability estimated at 32–39%, FVC at 40–41%, and FEV_1_/FVC at 46% 19, 20. Prediction accuracy of a trait approaches trait heritability, when that accuracy is measured in r^2^ or variance explained 21. However, prediction of >90% area under the receiver‐operating characteristic curve (AUC) is achievable while explaining roughly 30% of the variance and 40% of the heritability 22. For example, using the Welcome Trust Case Control Consortium (WTCCC) data and diseases 23,which have heritabilities estimated between 60% and 76%, maximum theoretical AUCs from models using only SNPs range from 93% to 99% 24.

In WGP many hundreds or thousands, or even all, available SNPs are used in a machine‐learning or regression‐based methodology agnostic to their previous associations or lack thereof to the phenotype of interest. This is an idea that has gained interest since genetic prediction based on smaller numbers of robustly associated SNPs has met with an inability to explain very much of the observed variation or heritability 25, 26. Growing evidence suggests that many complex disorders have polygenic etiologies, based on the small effects of many thousands of genetic variants 27. Methodologies that attempt to predict disease risk based on the combined effects of many SNPs are potentially able to exploit this genetic architecture 28.

Some authors have obtained significant prediction of disease or quantitative traits using only a small group of previously identified SNPs, including efforts predicting body mass index (57.4% AUC with 12 SNPs 29), type 2 diabetes (60% AUC with 18 SNPs 30), and others reviewed in Kundu et al. 31. Recent successes in WGP have resulted in much higher accuracies, include the prediction of celiac disease (90% AUC in replication cohorts 22), oral mucositis (82% accuracy 32), and skin cancer risk (64% AUC 33). Furthermore, several different authors have demonstrated accuracy (60–90% AUC) of a variety of WGP methodologies on the WTCCC diseases (bipolar disorder, Crohn's disease, coronary artery disease, hypertension, rheumatoid arthritis, type 1 and type 2 diabetes) 23, 34, 35, 36.

Predicting asthma incidence in young children using a set of 215 candidate SNPs for prediction has met with limited success (54% AUC 37). Using WGP to predict childhood asthma incidence resulted in an AUC of 54% using between 10,000 and 215,000 SNPs in a simple regression model; although prediction of childhood wheeze was more accurate (AUC 58%) 38. Others have tried to predict asthma‐associated phenotypes such as the bronchodilator response (BDR, change in pre‐ and post‐bronchodilator administration FEV_1_) among asthmatics, but only with two candidate SNPs 39. Prediction of reduced FEV_1_ was attempted with WGP methods, without success (AUC ∼52%) 38. Asthma exacerbations were significantly predictable with roughly 300 SNPs (66% AUC) 40.

We had two main goals in this work: (1) to determine whether WGP could accurately predict several asthma‐related phenotypes (pre‐ and post‐bronchodilator FEV_1_, airway hyper responsiveness, serum IgE, eosinophils, longitudinal lung function growth patterns [reduced growth and/or early decline vs. normal] and steroid responsiveness); and (2) to compare different WGP methodologies to identify the most powerful and accurate methods for prediction of asthma‐related phenotypes. We hypothesized that WGP could be successfully applied to asthma‐related phenotypes of clinical interest in a well‐phenotyped cohort. We further hypothesized that reduced growth of FEV_1_ and early decline of FEV_1_ are traits with high heritability, and thus can be well‐predicted from WGP methods. We applied several WGP methods to the Childhood Asthma Management Program (CAMP) cohort of mild‐to‐moderate persistent asthmatics, and demonstrated the utility of prediction based on SNPs to a number of outcomes within CAMP. Theoretical results suggest that these phenotypes are predictable with comprehensive, accurate genotyping in proportion to the heritability of these traits 14, 21; and we provided estimates of the genetic heritability for each of these traits. We also demonstrated increased prediction with the inclusion of relevant clinical and demographic covariates.

## Methods

### CAMP

We used data from the Childhood Asthma Management Program (CAMP) 41, 42, a study containing genome‐wide SNP data on 832 unrelated children with mild‐to‐moderate asthma enrolled in a randomized clinical trial at ages 5–12. Genotyping was previously performed at the Channing Division of Network Medicine using Illumina Quad 610 microarray chips (Illumina, Inc., San Diego, CA). Genotype data were filtered for quality by limiting investigation to autosomal SNPs with a minor allele frequency of at least 0.05 and probability of Hardy–Weinberg equilibrium of at least 0.001, using the PLINK2 software 43. This resulted in 455,481 SNPs available for WGP and heritability computation.

We selected a number of relevant asthma‐related phenotypes collected at baseline in the CAMP study: serum total IgE, eosinophil count (EOS), pre‐ and post‐bronchodilator FEV_1_ (percent‐predicted based on age, sex, height, race), bronchodilator response (BDR, (post‐FEV_1_ − pre‐FEV_1_)/pre‐FEV_1_), airway hyperresponsiveness (AHR, natural log of methacholine concentration needed for 20% reduction in FEV_1_), steroid responsiveness endophenotype (SRE, as described by Clemmer et al. 44); and longitudinal lung growth patterns 3: Normal Growth only (NG), Normal Growth with Early Decline (NG‐ED), Reduced Growth only (RG), Reduced Growth with Early Decline (RG‐ED), Early Decline irrespective of normal or reduced growth (ED‐All), and Reduced Growth with or without early decline (RG‐All). Lung growth patterns were observed based on longitudinal follow‐up of 12–16 years in CAMP continuation studies, while the other phenotypes were measured at or near randomization. Lung growth patterns were identified based on smoothed pre‐FEV_1_ spirometry performed at least annually as follows: normal growth was identified for subjects who were predominately above the 25th percentile of FEV_1_ for their age, sex, height, and race/ethnicity; reduced growth was indicated for subjects bellow the 25th percentile. Early decline was indicated for subjects at least 23 years of age at the conclusion of follow up who demonstrated a premature reduction from peak FEV_1_ achieved at the end of adolescence or early adulthood. More details of the longitudinal lung function phenotypes are available in McGeachie et al. 3.

Assessment of these baseline phenotypes has been previously described 41, 42. Briefly, total serum IgE and peripheral blood total eosinophil counts were measured by the radioimmunosorbent assays from blood samples collected during the screening sessions of CAMP. IgE and eosinophil counts were considered at log10 scale. Spirometry was performed according to ATS specifications. Baseline demographic and clinical covariates were also obtained, including age, sex, height, weight, body‐mass index (BMI), self‐reported race/ethnicity, CAMP treatment arm, and vitamin D. Vitamin D was measured as 25‐hydroxyvitamin D (25(OH)D) levels in serum collected at the time of enrollment using a radioimmunoassay 45. Missing values in data or covariates were imputed with the mean, to bias toward the null hypothesis of no association.

Additive genetic contribution to heritability if each phenotype was assessed using a genetic relatedness matrix 46. These heritabilities were computed for each phenotype based on all CAMP SNPs, using the GCTA software program 46, and following protocols described therein.

### Whole genome prediction

We then selected a number of polygenic prediction methods from the existing literature including naïve‐Bayes models, Least Absolute Shrinkage and Selection Operator (LASSO) regression 47, Support Vector Machines (SVM) 48, and a genetic relatedness matrix (GRM) method based on the “omic‐Kriging” method 34 and equivalent to the Genomic Best Linear Unbiased Predictor (G‐BLUP) 5. These methods were chosen for their previously demonstrated or theoretical suitability to prediction tasks including a very large number of variables. The naïve‐Bayes model includes all possible predictors, allowing each predictor to have a small effect on the posterior probability of the data 49, and is generally a simplified form of Bayesian regression 50. LASSO regression is a penalized logistic regression method that limits the number of non‐zero parameters used for prediction. SVMs compute class‐separating hyperplanes from kernel functions based on the dot‐products of predictors for each pair of subjects in the training set. GRM‐based prediction is similar: the GRM is a measure of similarity between subjects based on the dot products of their SNPs; then prediction of an unknown subject is achieved using the weighted sum of the known subjects’ classifications, with weights proportional to the genetic distance to the unknown subject. LASSO regression was implemented in the R statistical language using the glmnet package 51. All other methods were available or implemented in MATLAB (v R2014a, The MathWorks, Natick, MA), using standard MATLAB functions for SVMs and for naïve‐Bayes models. We used a simple linear kernel with the SVM. We implemented GRM‐based prediction de novo, following Wheeler et al. 34.

Although some methods can smoothly handle prediction in a continuous domain, to standardize our analysis we chose to convert continuous phenotypes to binary phenotypes for prediction by dichotomizing about the mean. To perform each prediction test, we first split the CAMP dataset into 75% training and 25% prediction populations. The 75% were used to train each prediction method, following Wu et al. 52 this was used for both variable selection (if applicable) and variable weighting (if applicable). The 25% were used to test each prediction method, from which we obtained AUCs 53 and convex‐hull AUCs 54. We used label‐permutation testing to obtain empirical *p*‐values for the difference between those AUCs and random guessing (AUC 50%). In this way, we tested in a statistically independent replication cohort. We then repeated this procedure 25 times and reported the average AUCs and *p*‐values obtained for each prediction method. For the GRM‐based prediction methods, we also used leave‐one‐out (LOO) validation, as follows. It is efficient to build the GRM on the entire cohort, but then hold out each participant in turn and use his/her relationship to the other cohort members to predict case/control status. This provided both a more robust model and more robust test of the GRM prediction methods, while retaining the essential independence of the test from the training data.

GRM‐based prediction methods can easily accommodate the inclusion of covariates in the prediction of unknown outcomes. We implemented the covariate GRM method suggested by Wheeler et al. 34 and performed prediction including covariates.

To compute the genetic relatedness matrix for use in the GRM‐based methods, we used a weighted sum of SNP differences as the genomic distance between two subjects 5, 55. This summation is typically performed with weights applied to SNPs based on the inverse‐variance of their allele frequency, which results in rarer alleles being weighted more heavily. However, any arbitrary weighting scheme can be used in place or in conjunction with these default weights. Herein, we considered weighting schemes based on the method of Croteau‐Chonka et al. 56 which prioritizes SNPs based on likelihood of functional significance according to demonstrated statistical association with gene expression levels (i.e., eQTLs), overlap with particular chromatin states, and high minor allele frequency. This method gave probability scores between 0% and ∼12% for each SNP. We considered three weighting schemes based on this score: W1 was based on the Croteau‐Chonka et al. scheme; W2 was based only on eQTLs and chromatin state, and not minor allele frequency; and a binary, thresholding, Non‐Zero Weight (NZW), wherein SNPs given zero weight in scheme W1 were removed from consideration in the WGP, resulting in a subset of 259,156 SNPs. The NZW SNP set was weighted according to the standard inverse‐variance SNP weights.

WGP methods were also tested upon the WTCCC datasets; these included the GWASes of 2000 cases for each of bipolar disorder (BD), Crohn's disease (CD), coronary artery diseases (CAD), rheumatoid arthritis (RA), type 1 diabetes (T1D), and type 2 diabetes (T2D), each one paired together with 3000 shared controls 23. We had previously processed and cleaned these data as described earlier 57. This resulted in approximately 375k SNPs in each of these six cohorts.

## Results

Characteristics of the CAMP cohort are reported in Table 1. Eight hundred thirty‐two CAMP participants were available, genotyped on 455,480 SNPs that passed quality control metrics. The cohort was composed of mostly non‐Hispanic white children, and also mostly male children. Average values of the 13 asthma‐related phenotypes investigated appear in Table 2.

**Table 1:** Demographic and descriptive statistics of the CAMP cohort, used as baseline covariates in the analysis. Measures were taken at or near randomization during the CAMP trial

	Mean (±s.d.)
Age	8.95 (2.13)
Age at diagnosis	3.08 (2.44)
Sex (N male, %)	505 (60.70%)
CAMP Treatment Group (N steroids, %)	252 (30.29%)
Race (N, %)	
Non‐Hispanic White	604 (72.60%)
African American	69 (8.3%)
Hispanic	96 (11.5%)
Asian/other	63 (7.6%)
Height (cm)	133.80 (13.82)
Body mass index (kg/m^2^)	18.22 (3.41)
Vitamin D, 25(OH)D	37.81 (15.60)

**Table 2:** The 13 asthma‐related phenotypes considered in this analysis. Airway hyperresponsiveness measured by provocative concentration of methacholine required to effect a 20% reduction in FEV_1_. Pre‐ and post‐bronchodilator FEV_1_ computed as percent predicted using age, sex, height, and race. Bronchodilator response computed as ((Post‐FEV_1_ − Pre‐FEV_1_)/Pre‐FEV_1_). Steroid responsiveness endophenotype computed following Clemmer et al. 44 Normal Growth, Reduced Growth, Early Decline, and related patterns were identified using longitudinal data according to McGeachie et al. 3

	Mean (±s.d.)
Airway hyperresponsiveness (AHR)	.07 (1.17)
Serum total IgE (log_10_) (IGE)	2.63 (.67)
Eosinophil count (log_10_) (EOS)	2.50 (.53)
Pre‐bronchodilator FEV_1_ (% predicted)	93.19 (14.20)
Post‐bronchodilator FEV_1_ (% predicted)	102.48 (12.76)
Bronchodilator response (BDR)	9.03 (7.44)
Steroid responsiveness endophenotype (SRE)	−.01 (1.29)
Normal growth only (N, %) (NG)	221 (26.56%)
Normal growth with early decline (N, %) (NG‐ED)	171 (20.55%)
Reduced growth only (N, %) (RG)	222 (26.68%)
Reduced growth with early decline (N, %) (RG‐ED)	177 (21.27%)
Early decline with normal or reduced growth (N, %) (ED‐All)	348 (41.83%)
Reduced growth with or without early decline (N, %) (RG‐All)	399 (47.96%)

The prediction accuracy of any genetic prediction methodology is ultimately limited by the heritability of that trait or phenotype in question 14, 24. In particular, the accuracy of the genomic relatedness matrix‐based methods are dependent upon the heritability explained by the SNPs assayed in the genome‐wide scan, upon which the GRM is constructed 55. Heritabilities were computed for each phenotype using the CAMP GWAS (Table 3). In general, the sample size we had available in CAMP (*n* = 832) is not sufficient to achieve accurate estimates of heritability using this method. However, some traits did have significantly high estimated heritabilities: Reduced Growth‐All (95%); both pre‐FEV_1_ and post‐FEV_1_ (81% and 83%); Normal Growth with Early Decline (55%); and IgE (53%).

**Table 3:** Additive Genetic Heritability of CAMP phenotypes. These are computed using the GCTA program 46, which does a computation based on the GRM to estimate the heritability explained by SNPs in a genome‐wide SNP sample in the cohort. For the 13 phenotypes, we have computed the heritability contained in the SNPs of the CAMP GWAS with and without adjustment for genotype principal components. Each heritability is listed with standard error in parentheses, where * indicates heritability significantly different from 0 at *p* < 0.05, and ** is significantly different from 0 at *p* < 0.01. NA: heritability estimate did not converge. Accuracy using the GRM method is listed in AUC, with ^+^ indicating prediction significantly greater than random (*p* < 0.05, permutation test)

	All SNPs (±Std)	All SNPs+PCs (±Std)	GRM (AUC, %)
AHR	NA	.45 (.29)	52.3
IGE	.42 (.24)**	.53 (.27)*	58.0^+^
EOS	.38 (.26)	.29 (.32)	54.1
Pre‐FEV_1_	.71 (.22)**	.81 (.22)**	58.1^+^
Post‐FEV_1_	.65 (.24)**	.83 (.22)**	56.1^+^
BDR	.52 (.25)	.67 (.24)*	53.6
SRE	.01 (.05)	.00 (.42)	51.1
NG	.54 (.22)**	.47 (.27)	59.6^+^
NG‐ED	.41 (.24)	.55 (.23)*	56.7^+^
RG	.38 (.25)	.49 (.26)	56.8^+^
RG‐ED	.25 (.20)	.17 (.27)	56
ED ALL	.25 (.20)	.22 (.28)	54.7
RG ALL	.94 (.19)**	.95 (.19)**	61.7^+^

**Figure 1:**
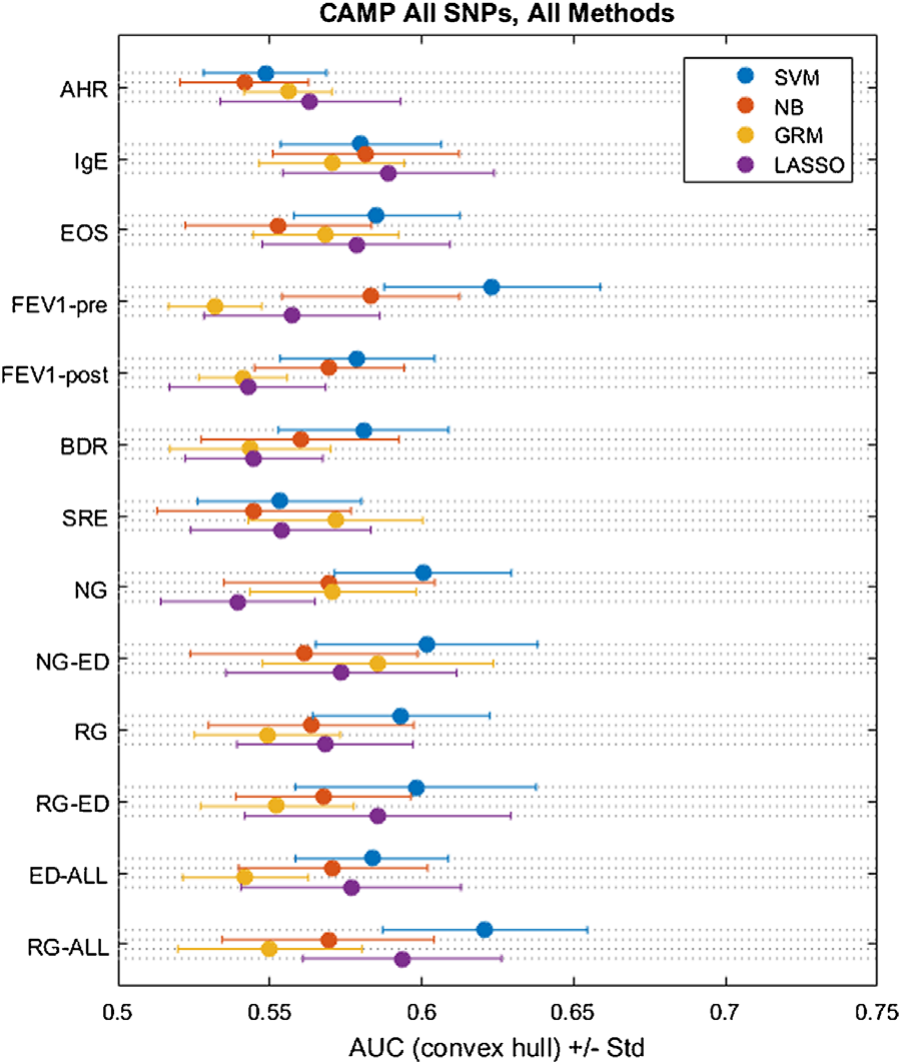
Asthma phenotypes predicted by four WGP methods. A number of WGP methods were used to predict 13 phenotypes in CAMP asthmatics. SVM, support‐vector machine; NB, naïve Bayes; GRM, genetic relatedness matrix; LASSO, least absolute shrinkage and selection operator regression; AHR, airway hyperresponsiveness; EOS, eosinophil count; Pre‐FEV_1_, pre‐bronchodilator forced expiratory volume in 1 sec; Post‐FEV_1_, post‐bronchodilator forced expiratory volume in 1 sec; BDR, bronchodilator response ((Post‐FEV_1_ − Pre‐FEV_1_)/Pre‐FEV_1_); SRE, steroid responsiveness endophenotype; NG, normal FEV_1_ growth (without early decline); NG‐ED, normal FEV_1_ growth with early decline; RG, reduced FEV_1_ growth (without early decline); RG‐ED, reduced FEV_1_ growth with early decline; ED All, early FEV_1_ decline (with normal growth or with reduced growth); RG All, reduced FEV_1_ growth (with or without early decline).

To assess the ability to predict each phenotype in proportion to its heritability, we performed Whole Genome Prediction in the entire CAMP cohort using four different methods (Fig. 1). We tested each method on each asthma phenotype for significant prediction above random (50% Area Under the Receiver Operator Characteristic Curve, AUC, is equivalent to random guessing) in the following way. We show mean convex‐hull AUC on the hold out test set, with error bars representing the standard deviation. We use mean *p*‐value for difference between random prediction (permutation test; label shuffling) to assess significance at a *p* < 0.05 threshold. Means are taken across 25 different random splits of the CAMP cohort into 75% training and 25% testing portions. We found significant prediction for none of the asthma‐related phenotypes using any of these unadjusted methods.

**Figure 2:**
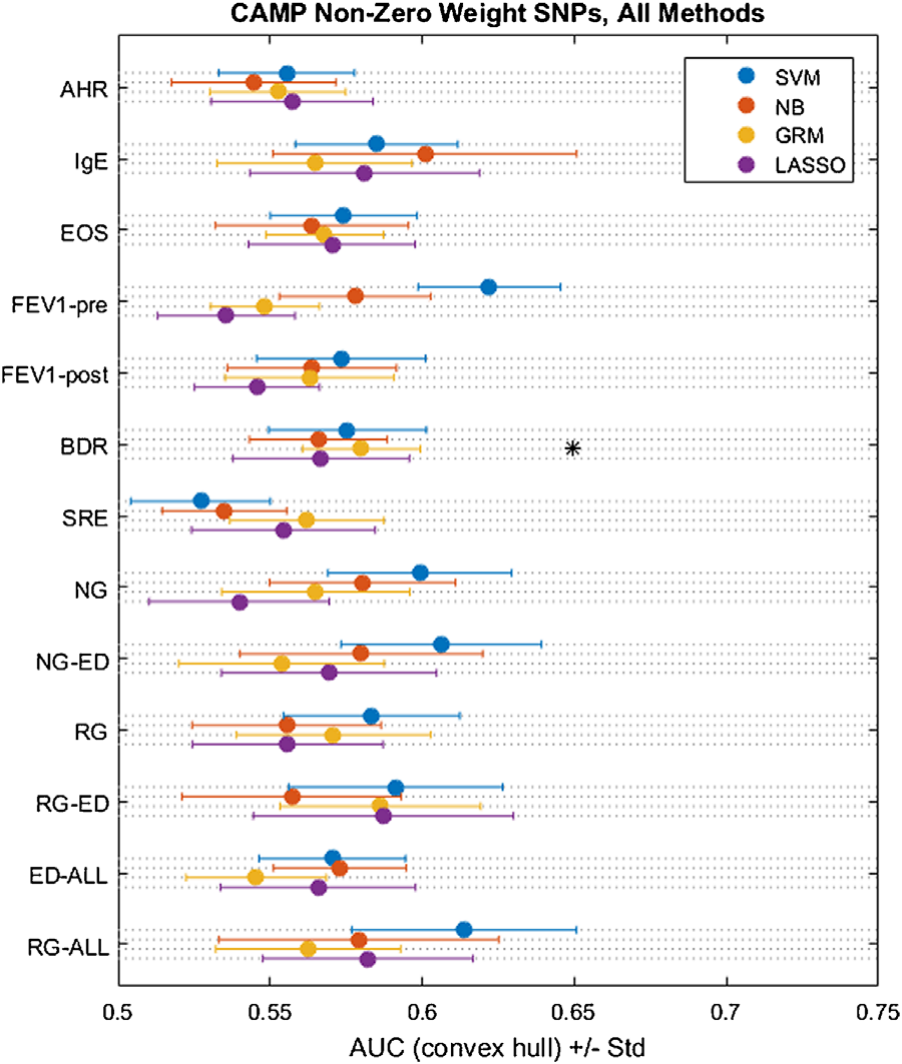
Asthma phenotypes predicted by four methods, using a reduced set of SNPs predicted to be of greater functional relevance. SVM, support‐vector machine; NB, naïve Bayes; GRM, genetic relatedness matrix; LASSO, least absolute shrinkage and selection operator regression; AHR, airway hyperresponsiveness; EOS, eosinophil count; Pre‐FEV_1_, pre‐bronchodilator forced expiratory volume in 1 sec; Post‐FEV_1_, post‐bronchodilator forced expiratory volume in 1 sec; BDR, bronchodilator response ((Post‐FEV_1_ − Pre‐FEV_1_)/Pre‐FEV_1_); SRE, steroid responsiveness endophenotype; NG, normal FEV_1_ growth (without early decline); NG‐ED, normal FEV_1_ growth with early decline; RG, reduced FEV_1_ growth (without early decline); RG‐ED, reduced FEV_1_ growth with early decline; ED All, early FEV_1_ decline (with normal growth or with reduced growth); RG All, reduced FEV_1_ growth (with or without early decline). *Indicate prediction meeting statistical significance for greater than random performance (AUC 0.50; *p* < 0.05, permutation test).

We additionally performed Whole Genome Prediction with genomic relatedness matrix‐based methods using several SNP‐reweighting schemes. We found improvements in prediction using the Non‐Zero Weight (NZW) SNP set (Supplemental Fig. S1). This set was composed of all SNPs given non‐zero weights by the procedure proposed by Croteau‐Chonka et al. 56 (see Methods), although retained SNPs’ weights were not changed from the inverse‐variance weights used by Yang et al. 46 These results are shown in Figure 2, where the GRM‐based method was able to significantly predict bronchodilator response.

To obtain tighter bounds on the performance of our GRM method, we used a Leave‐One‐Out (LOO) testing strategy. These results are shown in Table 3, and we found significant prediction for the GRM in a number of phenotypes (IgE, pre‐ and post‐FEV_1_, Normal Growth, Normal Growth with Early Decline, Reduced Growth, and Reduced Growth with or without Early Decline).

**Figure 3:**
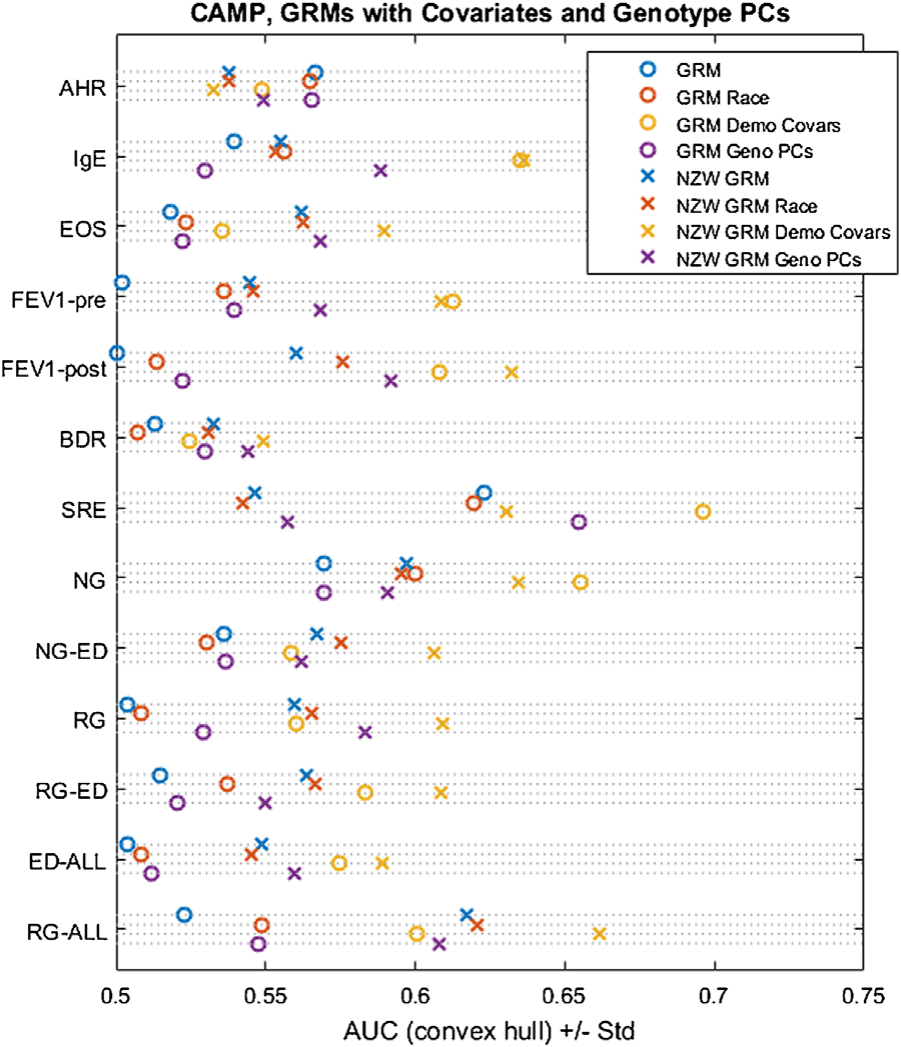
Prediction on CAMP cohort using GRMs with different covariates included, and a reduced set of Non‐Zero Weighted (NZW) SNPs. GRM, genetic relatedness matrix method using Leave‐One‐Out cross validation; AHR, airway hyperresponsiveness; EOS, eosinophil count; Pre‐FEV_1_, pre‐bronchodilator forced expiratory volume in 1 sec; Post‐FEV_1_, post‐bronchodilator forced expiratory volume in 1 sec; BDR, bronchodilator response ((Post‐FEV_1_ − Pre‐FEV_1_)/Pre‐FEV_1_); SRE, steroid responsiveness endophenotype; NG, normal FEV_1_ growth (without early decline); NG‐ED, normal FEV_1_ growth with early decline; RG, reduced FEV_1_ growth (without early decline); RG‐ED, reduced FEV_1_ growth with early decline; ED All, early FEV_1_ decline (with normal growth or with reduced growth); RG All, reduced FEV_1_ growth (with or without early decline). GRM‐only methods for IgE, EOS, post‐FEV_1_, NG, NG‐ED, RG, and RG‐All meet statistical significance for greater than random performance (AUC 0.50; *p* < 0.05, permutation test). Additionally, all combinations of the NZW GRM with clinical/demographic covariates were significant, except AHR and BDR.

The GRM‐based prediction method, as described by Wheeler et al. 34, allows easy integration of covariates into the predictive model. We included covariates for age, age of asthma diagnosis, sex, CAMP treatment group, height, body mass index, self‐reported race/ethnicity, and vitamin D serum level (Table 1), as well as the top six genotype principal components. In general, including covariates in the GRM models increased their accuracy (Fig. 3), with covariates resulting in improvements in prediction of all phenotypes except airway hyperresponsiveness (Fig. 3). In order to assess the possible effect of racial confounding, we included self‐reported race as a separate covariate with the GRM, finding that race alone did not increase the GRM's predictive ability (Fig. 3).

**Figure 4:**
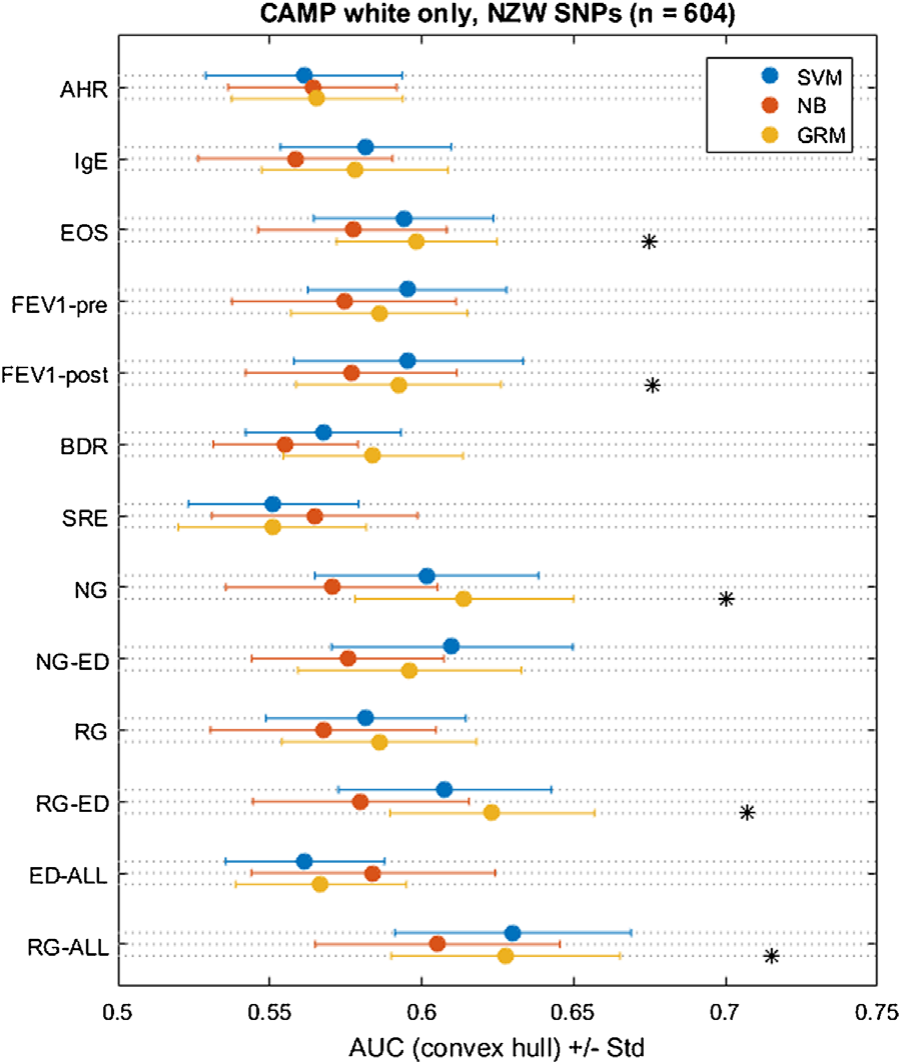
Prediction using WGP methods in CAMP non‐Hispanic white subjects only, with a reduced set of NZW SNPs. SVM, support‐vector machine; NB, naïve Bayes; GRM, genetic relatedness matrix; LASSO, least absolute shrinkage and selection operator regression; AHR, airway hyperresponsiveness; EOS, eosinophil count; Pre‐FEV_1_, pre‐bronchodilator forced expiratory volume in 1 sec; Post‐FEV_1_, post‐bronchodilator forced expiratory volume in 1 sec; BDR, bronchodilator response ((Post‐FEV_1_ − Pre‐FEV_1_)/Pre‐FEV_1_); SRE, steroid responsiveness endophenotype; NG, normal FEV_1_ growth (without early decline); NG‐ED, normal FEV_1_ growth with early decline; RG, reduced FEV_1_ growth (without early decline); RG‐ED, reduced FEV_1_ growth with early decline; ED All, early FEV_1_ decline (with normal growth or with reduced growth); RG All, reduced FEV_1_ growth (with or without early decline). *Indicate prediction meeting statistical significance for greater than random performance (AUC 0.50; *p* < 0.05, permutation test).

We also conducted WGP in the non‐Hispanic white subset of the CAMP cohort (*n* = 604). We observed similar prediction for most phenotypes and methods, but with increased significant predictions using the Genomic Relatedness Matrix and Non‐Zero Weight SNP set (Fig. 4). Eosinophils, post‐FEV_1_, Normal Growth, Reduced Growth with Early Decline, and Reduced Growth All were all significantly predicted using the GRM in non‐Hispanic whites. Results with other methods (Support Vector Machine and naïve Bayes) were non‐significant.

To test the generalizability of the Non‐Zero Weight SNP selection procedure, we compared performance in Welcome‐Trust Case Control Consortium datasets using the whole SNP set and the NZW SNP set (Supplemental Fig. S2). We found that the NZW strategy was helpful for the Genomic Relatedness Matrix method (but not naïve Bayes or Support Vector Machine), providing on average a 2.25% AUC increase (min 2.0%, max 2.8%) on each WTCCC disease.

## Discussion

Our main result was that the lung function growth patterns Reduced Growth and Early Decline are both conditions with strong genetic effects. We found that Reduced Growth‐All was predictable with the greatest AUC of any phenotype; additionally Reduced Growth with Early Decline and Reduced Growth only were predictable using a GRM and in the Non‐Zero Weight SNPs non‐Hispanic white subcohort, respectively. We additionally tested several other asthma‐related traits in childhood asthmatics, some of which displayed significant heritability. In conjunction with a SNP‐prioritizing scheme (i.e., Non‐Zero Weighting following Croteau‐Chonka et al. 56), GRM‐based methods achieved significant prediction on all phenotypes for which significant heritability was assessed (Table 3). Other methods of Whole Genome Prediction were not as effective.

Poor airway function is an important predictor of morbidity among asthmatics. Reduced Growth (RG), as defined here, refers to longitudinal lung function, measured by FEV_1_, over a period from childhood to young adulthood (9–26 years, on average), which is predominantly below the 25% percentile for a person of the same age, sex, race/ethnicity, and height. While this phenotype has not been similarly quantified, to our knowledge, in previous studies, much evidence shows that children with low lung function tend to remain at low lung function as they age and grow 58. Thus, while FEV_1_ and Forced Vital Capacity may only be ∼40% heritable, it is reasonable to think that low FEV_1_ has strong genetic components, which agrees with our heritability result. Furthermore, the very high heritability of the Reduced Growth pattern led directly to our highest prediction accuracy for a model including only SNPs on the Reduced Growth‐All phenotype. On the other hand, the Early Decline phenotype is very difficult to measure accurately, and was only described in a few previous works, and in those in association with smoking 59, 60. Our results, showing that a Genomic Relatedness Matrix can predict Early Decline, are important as both Reduced Growth and Early Decline can lead to chronic airway obstruction and even COPD 3. These are consistent with other studies of the heritability of lung function decline 18, and shows that Whole Genome Prediction can succeed in lung function despite the lack of replicable findings in genome‐wide association studies 61.

We included clinical and demographic variables as additional predictors with GRM‐based prediction; in many previous cases SNPs have been added to clinical and demographic factors in attempts to observe gains in prediction with genetic data 62, and this was true in our investigation as well. This is perhaps an indication that clinical and demographic data can be used to stratify subjects according to risk; or in a combined model directly with genotype to achieve the greatest possible accuracy.

We tested four major methods of Whole Genome Prediction, and found that LASSO and naïve Bayes were not successful predictors in this context. Genomic Relatedness Matrix‐based prediction and Support Vector Machine prediction did well in a number of tasks; with GRM method obtaining statistically significant prediction by our metric in a number of scenarios. The GRM is also fast and easily accommodates covariates, SNP weightings, and leave‐one‐out prediction; accordingly most of our subsequent analysis focused on the investigation of GRM‐based prediction.

Our results are limited by the application to the asthmatics in CAMP: one dataset with its own characteristics. These include inclusion criteria requiring mild or moderate persistent childhood asthmatics; and exclusion of both those with very mild asthma and those with severe asthma. While the measurement of accuracy with hold‐out test‐sets or cross‐validation schemes is typical in Whole Genome Prediction studies 22, 35, 63, the greatest test of these prediction methodologies is to apply them prospectively in an independent cohort; our results indicate that such test should result in significant prediction of a number of longitudinal spirometric phenotypes. Comprehensive longitudinal lung function pattern phenotypes are difficult to assess in additional cohorts, although it would be of great interest to investigate them further.

Genomic prediction of heritable asthma‐related clinical traits, such as reduced lung growth, may be possible purely with genetic information. The Non‐Zero Weight SNP selection criterion shows improvement in Genomic Relatedness Matrix‐based prediction in multiple cohorts. We also show that Whole Genome Prediction may reach clinical utility by combining demographic covariates with GRM‐based prediction. Longitudinal reduced growth is a phenotype with extremely high heritability.

## Conflicts of Interest

The authors report no conflicts of interest that could bias these results.

## Supporting information

Additional supporting information may be found in the online version of this article at the publisher's web‐site.

**Figure S1**. Comparison of four different SNP weighting schemes with GRM‐based prediction. GRM, weighted according to Yang et al. [55] default; GRM W1, weighted according to Croteau‐Chonka et al. [56]; GRM W2, weighted according to Croteau‐Chonka et al. [56], but without consideration of SNP minor allele frequency; GRM NZW, weighted according to Yang et al. [55], but with SNPs given zero‐weight by Croteau‐Chonka et al. [56] removed from consideration.
**Figure S2**. Whole Genome Prediction results on Welcome Trust Case Control Cohorts, also using the Non‐Zero Weight SNP set. BD, bipolar disorder; CAD, cardio vascular disease; CD, Crohn's disease; RA, rheumatoid arthritis; T1D, type 1 diabetes; T2D, type 2 diabetes; SVM, support vector machine; NB, naïve Bayes model; GRM, genomic relatedness matrix method.
