# Supplementary material for: Whole genome prediction and heritability of childhood asthma phenotypes
Source: Immun Inflamm Dis. 2016 Nov 28;4(4):487–96. doi: 10.1002/iid3.133 (PMC5134727; doi:10.1002/iid3.133)
Supplement: Supplementary file 1 — Figure S1. Comparison of four different SNP weighting schemes with GRM‐based prediction. GRM, weighted according to Yang et al. [55] default; GRM W1, weighted according to Croteau‐Chonka et al. [56]; GRM W2, weighted according to Croteau‐Chonka et al. [56], but without consideration of SNP minor allele frequency; GRM NZW, weighted according to Yang et al. [55], but with SNPs given zero‐weight by Croteau‐Chonka et al. [56] removed from consideration. Figure S2. Whole Genome Prediction results on Welcome Trust Case Control Cohorts, also using the Non‐Zero Weight SNP set. BD, bipolar disorder; CAD, cardio vascular disease; CD, Crohn's disease; RA, rheumatoid arthritis; T1D, type 1 diabetes; T2D, type 2 diabetes; SVM, support vector machine; NB, naïve Bayes model; GRM, genomic relatedness matrix method. [file IID3-4-487-s001.docx]

Whole Genome Prediction and Heritability of Childhood Asthma Phenotypes

Michael J McGeachie, PhD^1^

George L Clemmer, MS^1^

Damien C Croteau-Chonka, PhD^1^

Peter J Castaldi, MD^1^

Michael H Cho, MD^1^

Joanne E Sordillo, ScD^1^

Jessica A Lasky-Su, ScD^1^

Benjamin A Raby, MD^1^

Kelan G Tantisira, MD^1^

Scott T Weiss, MD^1^

^1^ Channing Division of Network Medicine, Brigham and Women’s Hospital and Harvard Medical School, Boston, MA.

Supplemental Material


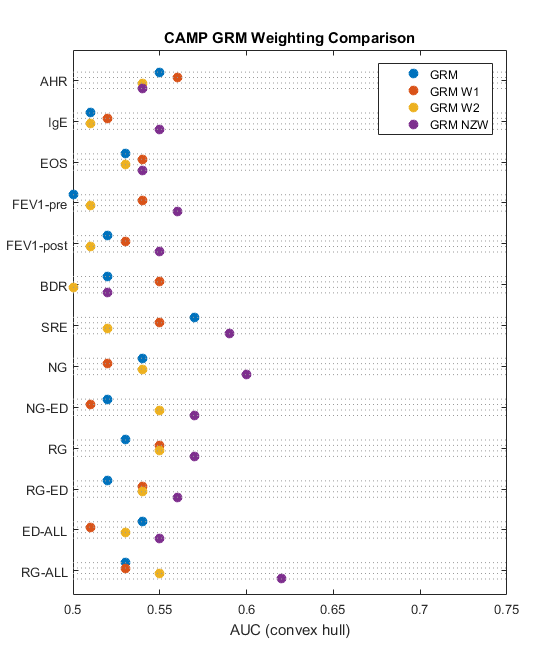


Supplemental Figure 1. Comparison of four different SNP weighting schemes with GRM-based prediction. GRM: weighted according to Yang *et al.*^55^ default. GRM W1: weighted according to Croteau-Chonka *et al*.^56^ GRM W2: weighted according to Croteau-Chonka *et al.*,^56^ but without consideration of SNP minor allele frequency. GRM NZW: weighted according to Yang *et al.,*^55^ but with SNPs given zero-weight by Croteau-Chonka *et al*.^56^ removed from consideration.


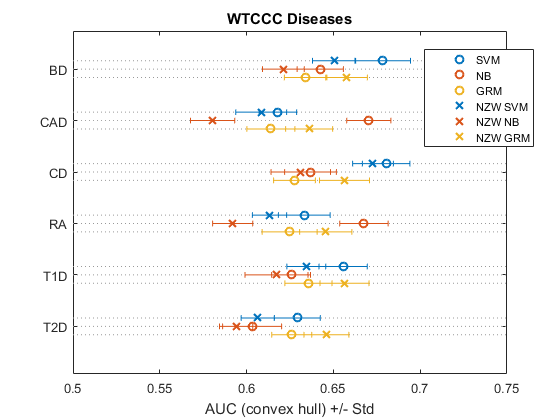


Supplemental Figure 2. Whole Genome Prediction results on Welcome Trust Case Control Cohorts, also using the Non-Zero Weight SNP set. BD = Bipolar Disorder, CAD = Cardio Vascular Disease, CD = Crohn’s Disease, RA = Rheumatoid Arthritis, T1D = Type 1 Diabetes, T2D = Type 2 Diabetes. SVM = Support Vector Machine, NB = Naïve Bayes model, GRM = Genomic Relatedness Matrix method.
